# Assisted suicide in patients with cancer☆

**DOI:** 10.1016/j.esmoop.2021.100349

**Published:** 2022-01-20

**Authors:** G. Montagna, C. Junker, C. Elfgen, A.R. Schneeberger, U. Güth

**Affiliations:** 1 Breast Service, Department of Surgery, Memorial Sloan Kettering Cancer Center, New York, USA; 2 Breast Center University of Basel, Basel, Switzerland; 3 Federal Statistical Office, Neuchâtel, Switzerland; 4 Breast Center Zürich, Department of Breast Surgery, Zurich, Switzerland; 5 University of Witten/Herdecke, Faculty of Medicine, Witten, Germany; 6 Psychiatric Services Grisons, Chur, Switzerland; 7 University of Zurich, Psychiatric Hospital, Zurich, Switzerland; 8 Albert Einstein College of Medicine, New York, USA; 9 University of Basel, Faculty of Medicine, Basel, Switzerland

**Keywords:** cancer, assisted suicide, assisted dying, end-of-life decision making, Switzerland

## Abstract

**Background:**

There are limited data on the long-term development and trends of assisted suicide (AS) among cancer patients.

**Patients and methods:**

Using data of the Swiss Federal Statistical Office, we analyzed AS trends over an 18-year period (1999-2016; total number of cases = 6553).

**Results:**

Among patients who underwent AS, cancer was the most common underlying disease (*n* = 2704, 41.3% of all AS cases). The most common cancer types were lung (14.0% of cancer-related AS cases), breast (11.0%) and prostate (10.1%). There was a slight preponderance of men compared with women (51.5% versus 48.5%). The proportion of AS cases within cancer types did not change over time. The ratio of cancer-related AS cases in relationship with all cancer-related deaths increased from 0.3% at the beginning of the study period (1999-2003) to 2.1% from 2014 to 2016 (change of age-standardized rates for men: +488%; for women: +417%). At the end of the study period (2014-2016), there were only minor differences between cancer-specific ratios, highest and lowest range: 1.1% (liver cancer) and 2.8% (breast, esophageal and lip/oral cavity/oropharynx cancer). Individuals who underwent AS because of cancer were considerably younger than those who choose AS on account of other diseases (73 years versus 80 years). The median age of people with cancer-related AS was similar to that of all cancer-related deaths (74 years): for women, the median age of cancer-related AS was 72, whereas for men it was 75. The median age at which AS took place increased over time.

**Conclusions:**

During the study period, the proportion of people who chose cancer-related AS has approximately sextupled. However, AS among cancer patients remains rare and represents only ∼2% of all cancer-related deaths.

## Highlights

• Cancer was the most common underlying disease for AS (41.3% of all AS cases).
• The ratio of cancer-related AS to all cancer-related deaths has sextupled over time: from 0.3% (1991-2003) to 2.1% (2014-2016).
• There were only minor differences between the cancer-specific ratios; in 2014-2016, ranges were between 1.1% and 2.8%.
• Individuals with cancer-related AS were younger than those who choose AS due to other diseases (73 vs. 80 years).
• The median age of people with cancer-related AS (73 years) was similar to that of all cancer-related deaths (74 years).

## Introduction

Over the last years, in most Western countries, there has been a clear and steady increase in the acceptance of the personal decision about the time of one’s own death,^1^, ^2^, ^3^, ^4^, ^5^ particularly for cases in which patients suffer from a terminal illness and/or experience unbearable and uncontrollable pain.^5^ Consequently, there is a growing number of countries where assisted suicide (AS) is legal under particular circumstances, including Switzerland, Belgium, the Netherlands, Luxembourg, Spain, Colombia, Canada, some US states (California, Colorado, Hawaii, New Jersey, Oregon, Washington state, Vermont and Washington, DC) and the Australian state of Victoria. Of these, Belgium, the Netherlands, Luxembourg, Canada and Colombia allow both euthanasia and AS.^6^

In countries where medical aid in dying is still illegal, there is an ongoing intensive, often controversial, debate on medical aid in dying, particularly regarding AS.^7^, ^8^, ^9^, ^10^, ^11^, ^12^, ^13^ Proponents see its legalization as an achievement of a modern society which places a high value on individuals’ autonomy. By contrast, opponents of assisted dying argue that these practices are fundamentally inconsistent with the physician’s professional role in which healing, managing pain and alleviation of suffering, but never intentionally inflicting death, are incontestable and non-negotiable cornerstones of medical practice.^12^ In addition, there is concern that if AS is legalized, safeguards put in place for these practices will be bypassed, which will lead to an uncontrolled extension to patients who are not terminally ill or do not suffer for severe symptoms (slippery slope argument).^12^

The views of the proponents and opponents of assisted dying are so diametrically opposed that mediation is hardly possible. However, public discussions about legalization of AS not only center on fundamental ethical matters. Public decision makers who decide whether medical aid in dying should be allowed in their countries might wonder what would happen if they did allow it. What long-term consequences will this bring? For example, how many people will take this course of action and from what medical conditions will they be suffering?

The primary aim of our work was to provide empirical data from Switzerland where AS has been legal for many years. Our goal was to describe how cancer-related AS has developed in a social context in which it is legal, socially acceptable, relatively freely available and where it clearly follows structured procedures.^14^^,^^15^ Using data from the Swiss Federal Statistical Office (FSO), we present the long-term development and trends of AS in Switzerland over an 18-year period (1999-2016), focusing on people who sought AS as a result of cancer. Particular consideration was given to the following questions:

• How high was the proportion of cancer patients among all AS cases?
• Among all cancer-related AS which type of cancer was more commonly seen?
• What proportion of cancer-related deaths happened due to AS?
• Compared with all patients who died of cancer, how old were patients who chose a cancer-related AS?
• Which developments and trends could be expected if there were to be a marked increase in cases of cancer-related AS over time?

Long-term empirical data regarding the highly complex religious and ethical–moral situation of assisted dying are scarce. Our analysis presents the longest period and largest number of cases ever reported on cancer-related AS in medical literature.

## Patients and methods

### Data source and procedure

The Swiss Cause of Death Statistics are based on medical cause listed on the death certificates. Diagnoses are recorded based on the International Classification of Diseases (ICD-10) and are collected by the FSO according to the rules defined by the World Health Organization (WHO).^16^ All collected data are treated anonymously and strictly confidentially and are subject to the provisions of the Federal Data Protection Act of 19 June 1992 (SR 235.1).^17^ Publications on the Cause of Death Statistics refer to persons who are resident in Switzerland (i.e. on the permanent resident population regardless of nationality and place of death).

Since the end of the 1990s, the FSO has received isolated notifications of AS. Because the ICD-10 does not have a specific code for AS, in the beginning these cases were classified as suicide by poisoning. Starting in 2009, AS has been coded consistently as X61.8 (i.e. as concomitant cause of death). The underlying cause of death was coded from the illness or disease leading to the request for assisted dying.^16^

For this study, we analyzed all death cases in Switzerland between 1999 and 2016.

### All death cases in Switzerland

During this 18-year period 1 127 864 people died (Table 1). Typical for an aging Western population, the median age at death was high at 82 years (men: 78 years, women: 85 years). In the study period, 288 955 cancer-related death cases were recorded. The proportion of deaths caused by cancer remained stable over time and approximated a quarter of all deaths (30% of the age-standardized mortality rate) (Table 1). People whose cause of death was cancer related were younger than those who died from a different nonaccidental cause (entire population: 74 versus 84 years; men: 74 versus 81 years; women: 75 versus 87 years).

**Table 1:** Death cases in Switzerland (1999-2016) with particular consideration of cancer and cancer-related AS as causes of death

Time period	Entire period 1999-2016	1999-2003	2004-2008	2009-2013	2014-2016	Change of ASR^a^ 2014-2016 versus 1999-2003
All death cases (men and women)	1 127 864	311 097	303 909	316 350	196 508	
Median age at death (5th-95th percentile)	82 (48-95)	80 (45-94)	81 (47-95)	82 (50-96)	83 (51-96)	
Cases with AS (% on all deaths)	6553	582 (0.2)	1161 (0.4)	2175 (0.7)	2635 (1.3)	
Median age of death (5th-95th percentile)	78 (51-93)	74.5 (43-91)	76 (50-92)	78 (52-93)	79 (54-93)	
- Cancer reported as underlying disease	2704	228	474	920	1082	
Median age at death (5th-95th percentile)	74 (51-90)	74 (47-88)	74 (48-88)	74 (52-91)	75 (52-90)	
- Other diseases	3484	282	556	1174	1472	
Median age of death (5th-95th percentile)	80 (51-94)	76 (39-92)	78 (48-93)	80 (50-94)	82 (55-94)	
- Unknown	365	72	131	81	81	
Median age at death (5th-95th percentile)	83 (59-94)	83.5 (54-94)	81 (57-94)	86 (63-95)	84 (66-93)	
Men						
All death cases	544 898	150 725	146 929	152 365	94 879	
Median age at death	78	77	78	79	79	
ASR		724.6	628.3	569.4	529.6	−27%
Cause of death: cancer (% on all-cause)	159 820 (29.3)	42 638 (28.3)	43 639 (29.7)	45 304 (29.7)	28 239 (29.8)	
Median age at death	74	73	74	74	75	
ASR (% on all-cause)		212.6 (29.3)	194.1 (30.9)	177.1 (31.1)	165.2 (31.2)	−22%
Cancer-related AS (% on all cancer-related deaths)	1393	114 (0.3)	230 (0.5)	484 (1.1)	565 (2.0)	
Median age at death	75	73	76	75	75	
ASR (% on cancer-related deaths)		0.6 (0.3)	1.1 (0.5)	1.9 (1.1)	3.3 (2.0)	+488%
Women						
All death cases	582 966	160 372	156 980	163 985	101 629	
Median age at death	85	84	84	85	86	
ASR (% on all-cause)		445.4	400.8	367.2	358.2	−20%
Cause of death: cancer (%)	129 135 (22.2)	34 188 (21.3)	35 299 (22.5)	36 660 (22.4)	22 988 (22.6)	
Median age at death	75	75	75	75	76	
ASR (% on all-cause)		125.3 (28.1)	119.7 (29.9)	110.8 (30.2)	108.9 (30.4)	−13%
Cancer-related AS (% on all cancer-related deaths)	1311	117 (0.3)	235 (0.7)	436 (1.2)	517 (2.2)	
Median age at death	72	67	71	73	73	
ASR (% on all cancer-related deaths)		0.5 (0.4)	0.9 (0.7)	1.4 (1.3)	2.6 (2.4)	+417%

AS, assisted suicide.

a ASR: age-standardized rate, defined as deaths per 100 000 person years, adjusted to the 2013 European Standard Population.

### All cases of AS

**Figure 1:**
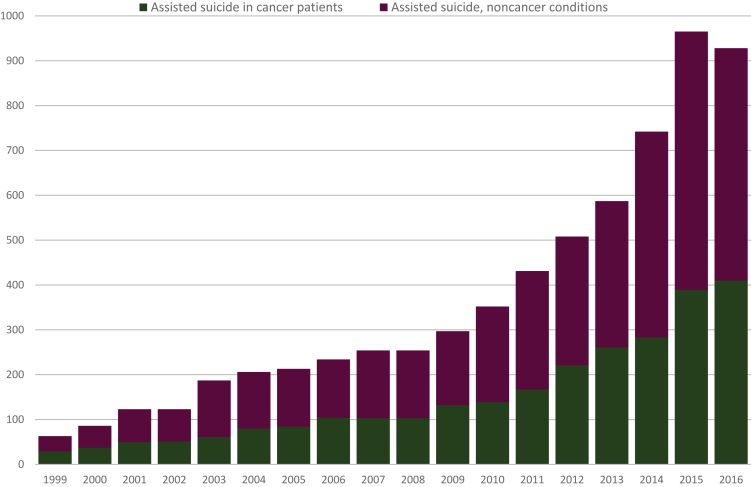
Development of the number of assisted suicide cases in Switzerland from 1999 to 2016.

Between 1999 and 2016 a total of 6553 cases of AS were registered among Swiss residents. During the observation period the number of AS cases rose substantially (Figure 1). The highest incidences occurred in 2015 (965 cases) and 2016 (928 cases). The percentage of AS from all death cases rose during the observation period from 0.2% (1999-2003) to 1.3% (2014-2016; men: 1.2%; women: 1.5%). AS was generally chosen by older people (median age 78 years) with a predominance of women (56.9%).

### Presentation of the data in four subperiods

For the purposes of the representation of the development of AS during the whole observation period of 18 years we have selected four periods (three 5-year-intervals and one 3-year interval: 2014-2016). The individual time intervals contain similar numbers of AS (1999-2003, range 63-187; 2004-2008; range 206-254; 2009-2013: range 297-587). For the last period a 3-year interval was deliberately chosen (2014-2016): in this period there was a further marked increase in AS of >100% compared with the preceding period. It appeared to be particularly important to represent this marked trend separately from the preceding years.

## Results

**Figure 2:**
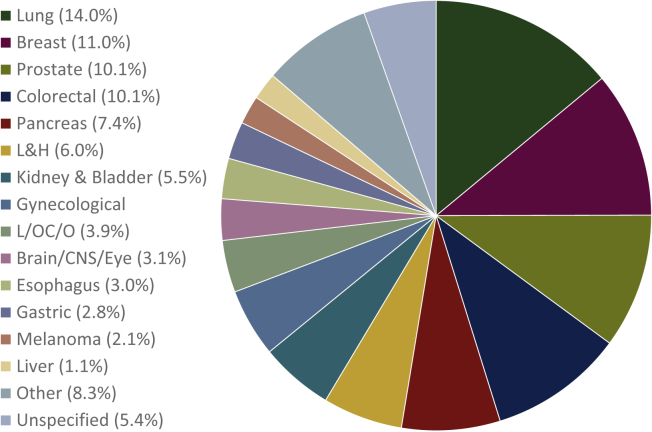
Cancer-related assisted suicide between 1999 and 2016 (*n* = 2704): distribution of different types of cancer. CNS, central nervous system; L&H, lymphatic and hematopoietic neoplasms; L/OC/O, lip/oral cavity/oropharynx.

Cancer was the most common underlying disease for AS (*n* = 2704, 41.3% of all AS cases). The proportion of cases in which a malignant tumor was the reason for AS remained stable over the four defined time intervals (39.2%, 40.8%, 42.3% and 41.0%, respectively; Table 1 and Figure 1). Figure 2 and Table 2 report AS cases in the 14 most common cancer types, which made up 22.7% of all death cases and 88.7% of all cancer-related deaths.

**Table 2:** AS in cancer patients in relation to individuals who died of cancer (Switzerland, 1999-2016)

Period	Entire period 1999-2016	1999-2003	2004-2008	2009-2013	2014-2016
Part 1. The five most frequent types of cancer (in 1, 4, 5: reported are the total number of cases and the data separated according to sex)					
**1. Lung cancer**					
Total number of lung cancer death cases	53 732	13 762	14 744	15 545	9681
(% of all death cases/all cancer death cases)	(4.8/18.6)	(4.4/17.9)	(4.8/18.7)	(4.9/19.0)	(4.9/18.9)
Median age at death (5th-95th percentile)	71 (51-87)	70 (49-86)	70 (50-86)	71 (51-87)	72 (53-88)
Number of AS cases (% of lung cancer-related death cases)	378	34 (0.3)	60 (0.4)	131 (0.8)	153 (1.6)
Median age at death (5th-95th percentile)	71 (53-88)	68 (53-85)	68 (51.5-89.5)	70 (52-90)	72 (53-87)
Men: number of lung death cases	35 923	9931	10 055	9923	6014
(% of all death cases/all cancer death cases)	(6.6/22.5)	(6.6/23.3)	(6.8/23.1)	(6.5/21.9)	(6.3/21.3)
Median age at death (5th-95th percentile)	71 (51-86)	70 (51-85)	70 (51-86)	71 (52-87)	72 (53-88)
Number of AS cases (% of lung cancer-related death cases)	185	25 (0.3)	24 (0.2)	55 (0.6)	81 (1.3)
Median age at death (5th-95th percentile)	72 (53-87)	69 (53-85)	68.5 (50-82)	75 (54-90)	73 (53-87)
Women: number of lung death cases	17 809	3831	4689	5622	3667
(% of all death cases/all cancer death cases)	(3.1/13.8)	(2.4/11.2)	(3.0/13.3)	(3.4/15.3)	(3.6/16.0)
Median age at death (5th-95th percentile)	70 (49-88)	69 (47-87)	70 (49-87)	70 (50-88)	71 (53-88)
Number of AS cases (% of lung cancer-related death cases)	193	9 (0.2)	36 (0.8)	76 (1.4)	72 (2.0)
Median age at death (5th-95th percentile)	69 (53-90)	66 (59-76)	67.5 (53-90)	69 (52-90)	70.5 (54-86)
**2. Breast cancer**^a^					
Total number of female breast cancer death cases	24 474	6683	6705	6921	4165
(% of all female death cases/all female cancer death cases)	(4.2/19.0)	(4.4/19.5)	(4.3/19.0)	(4.2/18.9)	(4.1/18.1)
Median age at death (5th-95th percentile)	72 (45-92)	71 (44-91)	71 (45-91)	73 (46-92)	74 (48-92)
Number of AS cases (% of breast cancer-related death cases)	296	29 (0.4)	50 (0.7)	100 (1.4)	117 (2.8)
Median age at death (5th-95th percentile)	72 (50-91)	65 (45-89)	71 (47-93)	73 (50-90)	75 (53-90)
**3. Prostate cancer**					
Total number of prostate cancer deaths cases	23 711	6590	6479	6698	3944
(% of all male death cases/all male cancer death cases)	(4.3/14.8)	(4.4/15.4)	(4.4/14.8)	(4.4/14.8)	(4.2/14.0)
Median age at death (5th-95th percentile)	81 (64-92)	80 (63-92)	81 (64-93)	82 (64-93)	82 (64-93)
Number of AS cases (% of prostate cancer-related death cases)	274	22 (0.3)	56 (0.9)	93 (1.4)	103 (2.6)
Median age at death (5th-95th percentile)	80 (63-93)	76.5 (59-91)	78 (65-90)	81 (64-93)	81 (64-93)
**4. Colorectal cancer (CR)**					
Total number of colorectal cancer death cases	29 603	8112	8061	8384	5046
(% of all death cases/all cancer death cases)	(2.6/10.2)	(2.6/10.6)	(2.7/10.2)	(2.7/10.2)	(2.6/9.9)
Median age at death (5th-95th percentile)	77 (52-92)	76 (53-91)	77 (52-92)	77 (52-92)	77 (52-92)
Number of AS cases (% of CR cancer-related death cases)	273	22 (0.3)	49 (0.6)	99 (1.2)	103 (2.0)
Median age at death (5th-95th percentile)	73 (48-90)	72 (50-84)	74 (50-88)	73 (48-91)	74 (45-91)
Men: number of colorectal cancer death cases	16 091	4249	4391	4655	2796
(% of all death cases/all cancer death cases)	(2.9/10.1)	(2.8/10.0)	(3.0/10.1)	(3.1/10.3)	(2.9/9.9)
Median age at death (5th-95th percentile)	75 (52-90)	74 (53-90)	75 (52-90)	75 (53-90)	76 (53-91)
Number of AS cases (% of CR cancer-related death cases)	141	11 (0.3)	19 (0.4)	58 (1.2)	53 (1.9)
Median age at death (5th-95th percentile)	73 (48-90)	78 (45-84)	70.5 (40-90)	73 (48-92)	75 (43-90)
Women: number of colorectal cancer death cases	13 512	3863	3670	3729	2250
(% of all death cases/all cancer death cases)	(2.3/10.5)	(2.4/11.3)	(2.3/10.4)	(2.3/10.2)	(2.2/9.8)
Median age at death (5th-95th percentile)	79 (52-93)	78 (53-92)	79 (52-93)	79 (52-93)	79 (52-93)
Number of AS cases (% of CR cancer-related death cases)	132	11 (0.3)	30 (0.8)	41 (1.1)	50 (2.2)
Median age at death (5th-95th percentile)	73.5 (50-91)	72 (50-84)	76 (53-88)	75 (54-89)	73 (47-93)
**5. Pancreatic cancer**					
Total number of pancreatic cancer death cases	19 065	4496	4887	5776	3906
(% of all death cases/all cancer death cases)	(1.7/6.6)	(1.5/5.9)	(1.6/6.2)	(1.8/6.8)	(2.0/7.6)
Median age at death (5th-95th percentile)	74 (53-90)	74 (52-90)	75 (52-90)	74 (53-90)	74 (53-90)
Number of AS cases (% of pancreatic cancer-related death cases)	200	9 (0.2)	39 (0.8)	61 (1.1)	91 (2.3)
Median age at death (5th-95th percentile)	72.5 (53-87.5)	71 (65-80)	73 (51-89)	73 (58-88)	71 (51-87)
Men: number of pancreatic cancer death cases	9222	2181	2334	2773	1934
(% of all death cases/all cancer death cases)	(1.7/5.8)	(1.5/5.1)	(1.6/5.4)	(1.8/6.1)	(2.0/6.8)
Median age at death (5th-95th percentile)	72 (51-88)	72 (52-88)	72 (51-87)	72 (51-88)	72 (52-88)
Number of AS cases (% of pancreatic cancer-related death cases)	98	3 (0.1)	22 (0.9)	36 (1.3)	37 (1.9)
Median age at death (5th-95th percentile)	72.5 (51-88)	72 (70-75)	79 (54-89)	73 (56-89)	71 (50-87)
Women: number of pancreatic cancer death cases	9843	2315	2553	3003	1972
(% of all death cases/all cancer death cases)	(1.7/7.6)	(1.4/6.8)	(1.6/7.3)	(1.8/8.2)	(1.9/8.6)
Median age at death (5th-95th percentile)	77 (54-91)	77 (53-91)	77 (54-91)	77 (54-91)	77 (54-91)
Number of AS cases (% of pancreatic cancer-related death cases)	102	6 (0.3)	17 (0.7)	25 (0.8)	54 (2.7)
Median age at death (5th-95th percentile)	72.5 (55-86)	69 (65-80)	67 (51-83)	77 (63-87)	72.5 (51-88)
Part 2. The 6-14 most frequent types of cancer					
**6. Lymphatic and hematopoietic neoplasms (LHNs)**					
Total number of LHN death cases	25 667	6833	6952	7222	4660
(% of all death cases/all cancer death cases)	(2.3/8.9)	(2.2/8.9)	(2.3/8.9)	(2.3/8.8)	(2.4/9.1)
Median age at death (5th-95th percentile)	76 (47-91)	75 (43-90)	76 (47-91)	77 (49-91)	78 (52-91)
Number of AS cases (% of LHN-related death cases)	162	16 (0.2)	17 (0.2)	56 (0.8)	73 (1.6)
Median age at death (5th-95th percentile)	78 (58-90)	79.5 (51-91)	77 (25-91)	76.5 (57-89)	78 (63-92)
**7. Kidney and bladder (K&B) cancer**					
Total number of K&B cancer death cases	16 309	4192	4405	4635	3077
(% of all death cases/all cancer death cases)	(1.4/5.7)	(1.3/5.5)	(1.5/5.6)	(1.5/5.7)	(1.6/6.0)
Median age at death (5th-95th percentile)	77 (55-92)	76 (54-91)	77 (54-92)	78 (55-92)	79 (56-92)
Number of AS cases (% of K&B cancer-related death cases)	149	14 (0.3)	16 (0.4)	48 (1.0)	71 (2.3)
Median age at death (5th-95th percentile)	77 (51-91)	78 (65-90)	70.5 (47-88)	80 (55-96)	78 (50-92)
**8. Gynecologic cancer**					
Total number of gynecooncologic death cases	14 370	3893	4023	3993	2461
(% of all female death cases/all female cancer death cases)	(2.5/11.2)	(2.4/11.4)	(2.6/11.5)	(2.4/10.9)	(2.4/10.7)
Median age at death (5th-95th percentile)	74 (49-91)	74 (48-91)	74 (49-91)	75 (49-91)	75 (49-91)
Number of AS cases (% of gynecologic cancer-related death cases)	139	14 (0.4)	27 (0.7)	40 (1.0)	58 (2.4)
Median age at death (5th-95th percentile)	68 (48-88)	63 (36-81)	70 (58-90)	68 (48.5-86)	70 (48-90)
**9. Cancer of lip, oral cavity, oropharynx (L/OC/O)**					
Total number of L/OC/O cancer death cases	6887	1766	1830	2026	1265
(% of all death cases/all cancer death cases)	(0.6/2.4)	(0.6/2.3)	(0.6/2.3)	(0.6/2.5)	(0.6/2.5)
Median age at death (5th-95th percentile)	66 (48-88)	64 (46-87)	66 (49-88)	67 (50-88)	69 (50-90)
Number of AS cases (% of L/OC/O cancer-related death cases)	106 (1.5)	5 (0.3)	24 (1.3)	42 (2.1)	35 (2.8)
Median age at death (5th-95th percentile)	70 (48-86)	61 (47-81)	66.5 (51-85)	71.5 (55-84)	71 (48-90)
**10. Brain, central nervous system (CNS), eye**					
Total number of CNS malignancy death cases	8730	2140	2387	2589	1614
(% of all death cases/all cancer death cases)	(0.8/3.0)	(0.7/2.8)	(0.8/3.0)	(0.8/3.2)	(0.8/3.2)
Median age at death (5th-95th percentile)	65 (32-85)	64 (31-84)	65 (31-85)	66 (33-86)	67 (33-87)
Number of AS cases (% of CNS malignancy-related death cases)	84 (1.0)	6 (0.3)	14 (0.6)	25 (1.0)	39 (2.4)
Median age at death (5th-95th percentile)	65.5 (40-83)	58.5 (40-77)	63 (26-83)	68 (37-85)	68 (43-88)
**11. Esophageal cancer**					
Total number of esophageal cancer death cases	7593	1951	2040	2218	1384
(% of all death cases/all cancer death cases)	(0.7/2.6)	(0.6/2.5)	(0.7/2.6)	(0.7/2.7)	(0.7/2.7)
Median age at death (5th-95th percentile)	71 (51-88)	70 (50-88)	70 (51-88)	71 (52-89)	72 (53-89)
Number of AS cases (% of esophageal cancer-related death cases)	82 (1.1)	8 (0.4)	13 (0.6)	22 (1.0)	39 (2.8)
Median age at death (5th-95th percentile)	73 (55-91)	75.5 (55-93)	67 (53-86)	73 (43-94)	73 (60-91)
**12. Gastric cancer**					
Total number of gastric cancer death cases	9828	2915	2674	2595	1644
(% of all death cases/all cancer death cases)	(0.9/3.4)	(0.9/3.8)	(0.9/3.4)	(0.8/3.2)	(0.8/3.2)
Median age at death (5th-95th percentile)	75 (47-91)	76 (48-91)	75.5 (46-91)	74 (47-90)	74 (49-91)
Number of AS cases (% of gastric cancer-related death cases)	76 (0.8)	12 (0.4)	17 (0.6)	21 (0.8)	26 (1.6)
Median age at death (5th-95th percentile)	74 (47-97)	72 (33-86)	72 (38-85)	78 (54-82)	71.5 (48-89)
**13. Melanoma**					
Total number of melanoma cancer death cases	5173	1171	1440	1572	990
(% of all death cases/all cancer death cases)	(0.5/1.8)	(0.4/1.5)	(0.5/1.8)	(0.5/1.9)	(0.5/1.9)
Median age at death (5th-95th percentile)	72 (41-91)	70 (38-90)	70 (40-90)	73 (42-91)	74 (47-91)
Number of AS cases (% of melanoma-related death cases)	58 (1.1)	5 (0.4)	13 (0.9)	20 (1.3)	20 (2.0)
Median age at death (5th-95th percentile)	74 (45-89)	77 (38-88)	66 (45-83)	73.5 (52.5-94.5)	75.5 (46-88)
**14. Liver cancer**					
Total number of liver cancer death cases	10 929	2529	2954	3258	2188
(% of all death cases/all cancer death cases)	(1.0/3.8)	(0.8/3.3)	(1.0/3.8)	(1.0/4.0)	(1.1/4.3)
Median age at death (5th-95th percentile)	72 (51-88)	72 (50-88)	72 (50-88)	72 (51-87)	73 (53-89)
Number of AS cases (% of liver cancer-related death cases)	55 (0.5)	1 (0.04)	5 (0.2)	26 (0.8)	23 (1.1)
Median age at death (5th-95th percentile)	70 (51-87)	56	77 (51-88)	68 (53-86)	70 (59-87)

AS, assisted suicide.

a We omitted male breast cancer cases. In the entire 18-year period, 136 men died on breast cancer (0.6% of all breast cancer death cases). There was only one case of a breast cancer-related AS (in the period 2004-2008; age at death: 67 years).

**Figure 3:**
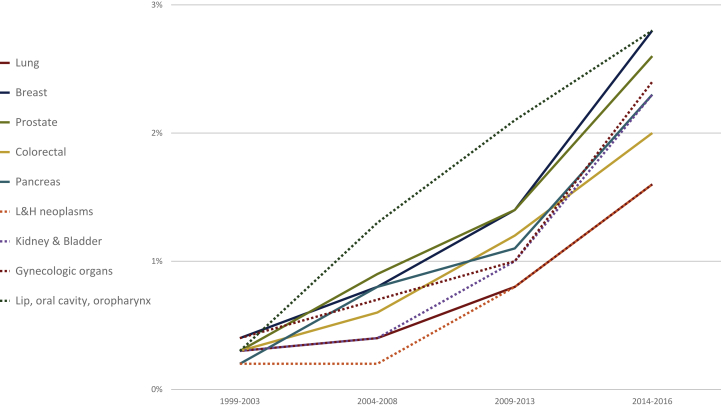
Development over time of the proportion of cancer-related assisted suicides to cancer-related deaths. Reported are the nine most frequent cancer types of cancer. L&H neoplasms, lymphatic and hematopoietic neoplasms.

Parallel to the substantial rise of the total number of AS cases, there was also a marked increase in cancer-related AS from 29 cases per year (in 1999) to 410 cases at the end of the study period (Table 1 and Figure 1). The crude ratio of cancer-related AS in relationship with all cancer-related deaths increased over time for all cancer types, ranging from 0.3% at the beginning of the study period (1999-2003) to 2.1% from 2014 to 2016 (age-standardized rate for men: 2.0%; change of age-standardized rates between 1999-2003 and 2014-2016: +488%; women: 2.4%, +417%; Table 1). There were only minor differences between the type-specific ratios in the most current study period (2016-2018): lower range: 1.1% for liver cancer, 1.6% for gastric and lung cancer and for lymphatic and hematopoietic neoplasms; upper range: 2.6% for prostate cancer and 2.8% for breast, esophageal and lip/oral cavity/oropharynx cancer (Table 2 and Figure 3).

Over time, AS increased for all cancer types. However, the proportion of AS cases within cancer types remained unchanged over time. This was true for all type of cancers, including the most common ones:

• Lung cancer, *n* = 378 (5.8% of all AS cases, range over the four observation periods: 5.2%-6.0%; 14.0% of cancer-related AS, range: 12.7%-14.9%),
• Breast cancer, *n* = 297 (4.5% of all AS cases, range 4.4%-5.0%; 11.0% of cancer-related AS, range: 10.8%-12.7%),
• Prostate cancer, *n* = 274 (4.2% of all AS cases, range 3.8%-4.8%; 10.1% of cancer-related AS, range: 9.5%-11.8%),
• Colorectal cancer *n* = 273 (4.1% of all AS cases, range 3.8%-4.6%; 11.0% of cancer-related AS, range: 9.5%-10.8%).

In cases of AS related to cancer there was a slight preponderance of men compared with women (51.5% versus 48.5%). Individuals who choose AS because of cancer were considerably younger than those who choose AS on account of other diseases (73 years versus 80 years). This is in line with the fact that overall the proportion of deaths due to cancer was higher for men than for women and that people who died because of cancer were younger than people who died on noncancer conditions (Table 1).

The median age of people with cancer-related AS (73 years) was similar to that of all cancer-related deaths (74 years). The median age of women with cancer-related AS was 72, whereas for men this was 75 years. For men, this was similar to the age of all cancer-related deaths (74 years); however, women with cancer-related AS were 3 years younger than those in the all cancer-related deaths group (75 years). For both sexes the age at which AS took place increased over the course of the study period (Table 2).

For most of the 14 cancer types analyzed, there were no substantial differences (defined as ≤2 years) between the age of cancer-related AS and the respective entire group of cancer-related deaths (Table 2, see entire periods). Individuals who chose AS due to colorectal cancer and women who did so due to gynecological cancers were younger than the respective entire groups of cancer-related death (gynecologic cancer: 68 years, –6 years; colorectal cancer: 73 years, –4 years). The median age of the individuals who chose AS due to lip/oral cavity/oropharynx cancer was 70, 4 years older than the entire group of death cases in this cancer type.

## Discussion

### The percentage of patients with cancer within the total group of people who chose AS (40%) were substantially lower than those in other countries where 70%-80% of assisted dying cases relate to cancer diseases^18^, ^19^, ^20^, ^21^

When comparing Swiss data with those from other countries where AS is legal,^3^^,^^6^^,^^14^^,^^22^^,^^23^ one must take into account that both different social and religious conditions as well as different frameworks of legal and professional conduct may exist. In particular, the situation in Belgium, the Netherlands, Luxembourg, Canada and Colombia is not directly comparable with that in Switzerland, because in these countries active euthanasia (i.e. the physician administers the lethal substance) is also allowed. This is not the case in Switzerland, where assisted dying implies that the doctor prescribes a patient a lethal substance or makes that substance available with the object of enabling the patient to commit suicide. The critical point, in contrast to active euthanasia, is that in AS the patients wishing to die must themselves carry out the last, decisive act of the procedure that will cause death.^24^ Usually, in Switzerland there is no doctor present during the process of dying; the terminal care is overtaken by the patient’s family and in most cases by a member of one of the organizations for assisted dying.

The low proportion of patients, with an underlying oncological disease, committing AS in Switzerland could reflect the greater extent of palliative care in oncology compared with other diseases. This would mean that oncological patients at the end of their journey can rely on good care and sufficient medical control of their symptoms.

It is, however, more likely that in a national and cultural context in which AS has a high level of social acceptance,^14^ many people, particularly the elderly, choose to assert their freedom to opt for assisted dying for reasons other than the ‘classical’ case of a late-stage cancer disease. The largest Swiss organization for assisted dying, ‘EXIT’, has propagated in recent years an easier access to ‘old age suicide’, which would include the AS of an elderly person who may not necessarily be suffering from a fatal disease but who, because of the sum of his or her current or expected complaints and ailments (reduction of bodily functions, decreasing sensory capacity and performance deficits) finds his or her quality of life seriously reduced.^25^ Suicidal tendencies in older persons usually result from several factors working in combination: chronic physical illnesses and disability, loss of control and autonomy, cognitive impairment, bereavement of loved ones and feelings of social isolation. Such factors lead to a high degree of hopelessness for the prospect of a satisfying future.^26^

### The group of people in which a cancer disease was the underlying cause for choosing AS was a predominantly geriatric group (median age of 73). The fear that the increase in the frequency of AS and its possibly uncritical spread will lead to a growing number of young people choosing this option does not seem to be the case in Switzerland. The opposite is in fact true, the median age has increased over the study period

The fact that cancer-related AS is an option predominantly chosen by elderly people implies that the aforementioned stress–diathesis model for old age suicide can probably also be applied to this group to some extent. In addition to the aforementioned age factors, a reduction in life expectancy may arise from the fact that some efficient oncologic therapies to control the progressive disease are more difficult to carry out in elderly patients. In some cases, patients fear the side-effects associated with the therapy and no longer believe themselves to be capable of undergoing such a therapy.^27^ Furthermore, for many elderly patients life satisfaction is closely related to self-rated health (as long as I am in good health), and less to diagnoses and more objective measures of health status.^28^ Retaining their current life circumstances and their quality of life plays a more significant role in determining their treatment options rather than aiming solely for an increase in survival time.^29^

### Despite a significant increase of AS over time (first observation period, 1999-2003, n = 50; most current observation period, 2014-2016, n = 400), the distribution of the underlying malignant disease did not change

During the 18-year period of our analysis we did not find that a particular cancer type became more associated with AS than others. The proportion of cancer-related AS cases to all cancer deaths remained roughly the same for each type of cancer (as did the distribution of the underlying diseases in the total group of AS). This stable distribution could be interpreted as an indication that the process in the terminal phase of the various illnesses is essentially similar.^30^

One could hypothesize that patients who suffer from slow-progressive cancer types have a higher likelihood of dying of AS (inversely phrased: that patients who suffer from rapidly progressing disease and thus do not have sufficient time for the decision-making process have a lower likelihood of assisted dying). Our data, however, cannot support these hypotheses because the differences in the proportion of AS cases within the different types of cancer are very similar; the difference between the type of cancer with the highest rate of AS and the one with the lowest rate during the most recent study period (2014-2016) was just ∼1.7% (1.1% for liver cancer versus 2.8% for breast, esophageal and lip/oral cavity/oropharynx cancer). Accordingly, we did not observe distinct differences in AS rates for cancer types with usually longer survival in the palliative situation (e.g. breast and prostate cancer) compared with cancer entities that usually lead to death more rapidly (e.g. pancreatic and gastric cancer).

### Limitations

The main limitations of this study are related to how the cause of death was recorded particularly at the beginning of the study period (e.g. lack of a central obligatory registry for AS, cases may be missed or not reported appropriately, death certificates might be an error-prone source for information about the underlying disease), which must be considered in the interpretation of our data. For more detailed information on these points, we refer to a recent overview from Steck and colleagues.^31^

### Conclusion

During the observation period the proportion of people who chose cancer-related AS has approximately sextupled. With a current share of 2% of all cancer-related deaths, it is, however, not justified to view this as a mass phenomenon.
